# Correction: USP7 facilitates brain tumor survival upon glucose deprivation by regulating phosphofructokinase muscle-type nuclear translocation in mice

**DOI:** 10.1371/journal.pbio.3003751

**Published:** 2026-04-07

**Authors:** The PLOS Biology Staff

The first two authors, Siyang Wu and Ruixiu Cao, should be noted as shared first co-authors on this work.

The publisher apologizes for the error.
